# Supplementary material for: Selenium nanoparticle loaded on PVA/chitosan biofilm synthesized from orange peels: antimicrobial and antioxidant properties for plum preservation
Source: BMC Chem. 2025 Aug 20;19(1):245. doi: 10.1186/s13065-025-01608-w (PMC12366351; doi:10.1186/s13065-025-01608-w)
Supplement: Supplementary file 1 — Supplementary Material 1 [file 13065_2025_1608_MOESM1_ESM.docx]

**Selenium nanoparticle loaded on PVA/chitosan biofilm synthesized from orange peels: Antimicrobial and Antioxidant Properties for Plum Preservation**

**Hamza T. O. Abdelaziz^1†^, Seif Eldin M. Mohamed^1†^, Samir K. A. Younis^1†^, Nada Ahmed^1†^, Mary N. Michaeel^1†^, Samah H. Abu-Hussien^2^, Ashraf Bakry^3^, Naglaa M. Ebeed^3^, Mohamed A. Nasser^4^, Mohamed K. Abou El-Nasr^4^, Mahmoud Adel^4^, Bahaa Hemdan^5*^, Mahmoud Salah^6^ and Salwa M. El-Sayed^7*^**

^1^Biotechnology Program, Faculty of Agriculture, Ain Shams University, Cairo,11241, Egypt

^2^Department of Agricultural Microbiology, Faculty of Agriculture, Ain Shams University, Cairo, 11241, Egypt

^3^Department of Genetics, Faculty of Agriculture, Ain Shams University, PO Box 68, Hadayek Shoubra, Cairo, 11241, Egypt

^4^Department of Horticulture, Faculty of Agriculture, Ain Shams University, Cairo,11241, Egypt

^5^Water Pollution Research Department, Environmental Research Division, National Research Centre, 33 El Buhouth Street, P.O. Box 12622 Cairo, Egypt

^6^Department of Environmental Agricultural Science, Faculty of Graduate Studies and Environmental Research, Ain Shams University, Cairo, 11566, Egypt

^7 6^Department of Biochemistry, Faculty of Agriculture, Ain Shams University, Cairo, 11241, Egypt

**^†^**Equal contribution authors

*Corresponding author:

Salwa M. El-Sayed ([Salwa_sedeek@agr.asu.edu.eg](mailto:Salwa_sedeek@agr.asu.edu.eg).

Bahaa Hemdan ([bahaa_nrc@yahoo.com](mailto:bahaa_nrc@yahoo.com)

**Abstract**

This study investigates the eco-friendly synthesis, characterization, and biological activity of selenium nanoparticles (SeNPs) incorporated into a polyvinyl alcohol/chitosan (PVA/CH) composite for antimicrobial, antioxidant, and food preservation applications. SeNPs were synthesized using orange peel extract as a reducing and stabilizing agent. Characterization through UV-Vis spectroscopy, FTIR, dynamic light scattering (DLS), TEM and zeta potential analysis confirmed the formation of stable, well-dispersed SeNPs. The antimicrobial activity of PVA/chitosan (PVA/CH) films incorporated with selenium nanoparticles (SeNPs) was evaluated against five pathogenic bacterial strains, showing a concentration-dependent enhancement, with the 1% SeNPs formulation achieving the highest inhibition zones across all tested pathogens. Se nanoparticles showed a more compact structure and excellent thermal and UV stability into PVA/CH-SeNPS film. Antioxidant potential was assessed using DPPH radical scavenging assays. The composites showed concentration-dependent free radical scavenging, with the highest activity observed at 1% SeNP concentration (IC50:45.2 µg/mL), closely approaching that of ascorbic acid (IC50: 38.6 µg/mL). HPLC analysis of orange peel extract identified high levels of rutin (546.46 mg/kg), quercetin (217.45 mg/kg), and chlorogenic acid (72.75 mg/kg), which likely contributed to the observed antioxidant properties. Cytotoxicity tests using Vero cells demonstrated high biocompatibility, with over 80% cell viability at all tested concentrations, confirming the composites’ potential for safe biomedical applications. Additionally, the use of PVA/CH-SeNP composites as an edible coating for Hollywood plum (Prunus domestica L. cv. Hollywood) was evaluated to extend postharvest storage. Fruits coated with 0.5% and 1% PVA/Ch-SeNPs showed significantly reduced weight loss (3.64% and 2.84%, respectively, compared to 6.91% in the control) and maintained higher firmness (7.20 N/cm² vs. 4.12 N/cm² in the control). The coatings also increased total soluble solids (TSS: 14.6°Brix), decreased acidity (0.936%), and enhanced L-ascorbic acid (19.86 mg/100g) and anthocyanin content. These results highlight the potential of PVA/CH-SeNP composites as multifunctional biomaterials for antimicrobial, antioxidant, and food preservation applications. Future studies should explore their in vivo efficacy, long-term stability, and potential for large-scale applications.

**Keywords:** Green synthertized SeNPs, PVA/Chitosan composite, Antimicrobial activity, Antioxidant properties, Edible coating, food security

**Introduction**

Nanoparticles are tiny and have a large surface area compared to their volume, which gives them benefits like better reactivity, stability, and availability in the body, making them important for uses that need precise interactions at the molecular or cellular level [2]. Selenium, an essential micronutrient, plays a crucial role in various biological functions, including antioxidant defense, immune function, and thyroid hormone metabolism [1]. Selenium nanoparticles (SeNPs) have gained significant attention recently due to their unique properties, particularly in biomedical, environmental, and material science applications. In its nanoparticle form, SeNPs exhibit enhanced biological activity compared to its bulk counterpart, which could be utilized in medicine, agriculture, and environmental protection. SeNPs have shown promise in areas such as cancer treatment, antimicrobial therapy, and antioxidant applications. The ability of SeNPs to scavenge reactive oxygen species (ROS) and protect cells from oxidative stress has positioned them as potent antioxidants [3]. This property is particularly valuable in the context of preventing diseases linked to oxidative stress, including cancer, cardiovascular diseases, and neurodegenerative disorders [4]. SeNPs exhibit broad-spectrum antimicrobial activity against both gram-positive and gram-negative bacteria. Their mode of action is believed to involve the generation of ROS, disruption of microbial cell membranes, and interference with microbial metabolism, which leads to bacterial cell death [5]. The ability to inhibit the growth of pathogenic bacteria makes SeNPs an attractive candidate as an antimicrobial agent utilized in agricultural and environmental applications [6].

The synthesis of SeNPs can be achieved through various chemical, physical, and biological methods [7]. Green synthesis methods based on plant extracts offer a more sustainable and environmentally friendly approach, which resulted in the production of biocompatible nanoparticles [8]. Plant extracts have been extensively studied for their ability to reduce selenium ions to SeNPs while simultaneously acting as stabilizing agents to prevent nanoparticle aggregation. These extracts, rich in phytochemicals such as flavonoids, phenolics, and terpenoids, contribute to the biological activity of the synthesized nanoparticles, enhancing their antioxidants and antimicrobial properties [9]. Moreover, incorporating SeNPs into polymer matrices has emerged as a promising strategy to further enhance their stability and functionality [10]. Polyvinyl alcohol (PVA) and chitosan (CH) have been widely used as nanocarriers due to their biocompatibility, mechanical strength, and ability to form films and hydrogels. [11]. CH has garnered attention for its antimicrobial and wound-healing properties, making it an ideal candidate for biomedical applications. PVA, on the other hand, is a synthetic polymer known for its excellent film-forming ability, water solubility, and chemical resistance [12]. Therefore, the combination of PVA and chitosan provides a robust matrix for the stabilization of SeNPs, allowing for controlled release and prolonged activity for postharvest treatment [13].

We aimed to study the loading efficiency of SeNP in PVA/CH as an edible biofilm in terms of enhancing its antimicrobial, antioxidant, and cytotoxic properties in vitro. Additionally, the effect of the PVA/CH-SeNP composite on the quality and shelf life of Hollywood plum (*Prunus domestica* L. cv. Hollywood) was investigated in vivo.

**Materials and Methods.**

**Materials**

All chemicals used for the experiment including sodium selenite (purity of 99.0%), acetic acid, ethanol, Polyvinyl Alcohol (PVA) (purity of 99.0%), chitosan, 1,1-diphenyl-2-picrylhydrazyl (DPPH) were purchased from Sigma Aldrich, Germany. Deionized water was used in all experiments.

**Microorganisms**

The antimicrobial activity of the nanoparticles was tested against five pathogenic bacterial strains: *Escherichia coli* ATCC 8379, *Klebsiella pneumoniae* ATCC 00607, *Salmonella typhi* DSM 17058, *Bacillus cereus*, and *Staphylococcus aureus*. These strains were obtained from MIRCEN, Faculty of Agriculture, Ain Shams University. All strains were maintained on glucose agar (Difco Manual, 1984) and stored at 4°C.

**Plant material**

Uniform Hollywood plum (*Prunus domestica L. cv. Hollywood*) fruits were purchased from private farm on Cairo-Alexandria Desert Road in the Khatatba area, Egypt, harvested at the physiologically mature stage, characterized by full skin coloration and a total soluble solids (TSS) value of 10. Fruits were selected for uniformity in shape, weight, and color, ensuring the absence of visible pathogen infection. Surface sterilization was performed by immersing the fruits in a 1% sodium hypochlorite solution for three min, followed by rinsing with clean water and air-drying at room temperature. The fruits were assigned to five treatments: control, PVA/CH, PVA/CH-SeNPs at 0.25%, PVA/CH-SeNPs at 0.5%, and PVA/CH-SeNPs at 1%. They were packed in perforated carton boxes and divided into two groups per treatment. One group was used to assess weight loss, while the other was used for periodic evaluation of physical and chemical properties. Each treatment was replicated six times, with each replicate consisting of 10 fruits per box. The fruits were stored at 0 ± 2°C and 90 ± 5% relative humidity for four weeks.

**Preparation of orange peels extract.**

Orange peels were washed with tap water to remove impurities, cut into small pieces, and 5 g were blended with 150 mL of deionized water in a blender (mixer) at 1000 rpm for 3 min at room temperature, followed by the extract orange peels being centrifuged for 5 min at 6000 rpm. The resulting orange peel extract (OPE) was stored at 4°C for further use.

**Separation and determination of phenolic compounds of orange peels by High-Performance Liquid Chromatography (HPLC).**

Using an Agilent 1260 HPLC system with an Eclipse C18 column, a 0.2g sample of fresh orange peels was extracted using HPLC-grade methanol, centrifuged, and examined. The mobile phase included water (A) and 0.05% trifluoroacetic acid in acetonitrile (B), at a flow rate of 0.9 mL/min. A linear gradient elution was used, escalating from 60% A (8–12 min) to 82% A (12–20 min), facilitating effective isolation and quantification of phenolic components in the orange peel extract [14].

**Determination of vitamin C content of orange peels by HPLC**.

The detection of ascorbic acid in orange peels was performed using HPLC according to [15]. A 10 g sample of orange peels was dissolved in an o-phosphoric acid solution, vortexed, and then centrifuged. The supernatant was subjected to filtration and then evaluated using HPLC. The Agilent 1260 Infinity HPLC system equipped with a HyperClone™ BDS C18 column was used. Separation was accomplished with a binary linear gradient of sodium dihydrogen phosphate and methanol. A 20 µL sample was injected, and detection was performed at 270 nm with a VWD detector.

**Synthesis of selenium nanoparticles (SeNPs) using OPE.**

SeNPs were synthesized by reducing selenium ions in sodium selenite (Na_2_SeO_3_) using fresh OPE extract as a reducing agent. OPE was used in the synthesis of SeNPs. Fresh OPE (10 mL) was combined with 90 mL Na_2_SeO_3_ solutions (0.2 mM), then, the mixture was homogenized for 2 h on a magnetic stirrer at room temperature. A distinct visual change occurred during the process, with the reaction mixture transitioning from yellow to a noticeable red color, signaling the reduction of selenite and the formation of Se-NPs. Further the solutions were kept on an orbital shaker and observed at an interval of 24 h. After this color change, the synthesized SeNPs were purified by centrifugation at 6000 rpm for 20 min. These samples were further characterized and subjected to preparation of PVA/CH-SeNPs composite packaging [16].

**Preparation of PVA/CH-SeNPs composite films.**

The PVA/CH was made by chitosan solution was prepared by dissolving 5 g chitosan in 100 mL acetic acid solution (1%) and stirred overnight using a magnetic stirrer until to obtain a homogeneous phase. In the same way, a PVA solution (5 % w/v) was prepared under continuous stirring at 60°C. Then, both solutions were mixed at ratio 1:1 (v/v) at room temperature under continuous agitation [17]. Then, prepared composite nanofilms from PVA/CH-SeNPs by slowly adding the required concentrations of nano-selenium (SeNPs) (0.25, 0.5, and 1%) to mixture of PVA/CH solution, and they were mixed well for 15 minutes on a magnetic stirrer at room temperature and pH 4.6 before pouring into a Petri dish. We prepared PVA/CH-SeNPs nano-composite films have been created using solution casting technique; the solution of each sample was poured into a Petri dish and dried in the furnace at room temperature for 3 days. The thickness of prepared films was in the range of 0.2 mm **(see Figure 1).**

**Figure 1:** Synthesis of selenium nanoparticles (SeNPs) using OPE and preparation of PVA/CH and PVA/CH-SeNPs composite films.

**Characterization analysis of the prepared SeNPs and PVA/CH-SeNPs nano-composite films**

Advanced analytical techniques comprehensively characterized SeNPs, PVA/CH and PVA/CH-SeNPs nano-composite films. UV-Visible spectroscopy employed UV Analyst-CT 8200 spectrophotometer (200-800 nm) to evaluate optical properties. Fourier Transform Infrared Spectroscopy (FTIR) used Shimadzu Tracer-100 (1000-4000 nm, 4 cm⁻¹ resolution, refractive index 2.4) to analyze chemical composition. Dynamic Light Scattering (DLS) utilized Zetasizer Nano Series (HT), Nano ZS, Malvern Instruments (UK) to measure particle size distribution. Zeta potential analysis was performed using NICOMPTM 380 ZLS Analyzer, employing phase analysis light scattering with 286 kHz count rate, wavelength 1.54 nm at 30°C. Transmission Electron Microscopy (HR-TEM, JEOL JEM-2100, 200 kV, 2838 cps) provided high-resolution imaging of nanoparticle structure [18].

**Stability of PVA/CH-SeNPs nano-composite film**

**Effect of pH on PVA/CH-SeNPs nano-composite film**

The effect of pH on PVA/CH-SeNPs nano-composite film was investigated. The pH of the PVA/CH-SeNPs nano-composite solution was adjusted to 3, 7, and 12. Then, Dynamic Light Scattering (DLS) utilized Zetasizer Nano Series (HT), Nano ZS, Malvern Instruments (UK) to measure particle size distribution [19].

**Effect of UV exposure time on PVA/CH-SeNPs nano-composite film**

The PVA/CH-SeNPs nano-composite film was prepared in transparent glass bottles and placed in an ultraviolet (UV) light cabinet (power 40 W, wavelength 254 nm UV-C radiation) which was situated at 20 cm over the PVA/CH-SeNPs nano-composite film. The stability of PVA/CH-SeNPs nano-composite was investigated by measuring the retention rate of Se after 0, 1, 2, 3, and 4 h of UV exposure [19]. The SeNPs were extracted from PVA/CH-SeNPs nano-composite film using deionized water, and then the extracts were centrifuged at 6000 rpm for 10 min. The Se was measured using an Inductively coupled plasma mass spectrometry (ICP-MS) [20]. The retention rate of Se was determined using the following equations:

$$\% Retention rate = \frac{The determined Se amount}{Total Se amount} X 100$$

**Effect of storage time PVA/CH-SeNPs nano-composite film.**

The freshly prepared PVA/CH-SeNPs nano-composite was stored incubator at 37 ^◦^C in a dark place for 0, 4, 8, 12, and 16 days . The SeNPs were centrifuged at 6000 rpm for 10 min, and the supernatants were used to determine the retention rate of selenium [19].

**Thermal stability of PVA/CH-SeNPs nano-composite film.**

Using the Netzsch DSC-200PC analyzer (Germany), the thermal stability of PVA/CH-SeNPs nano-composite assessment was completed. The of PVA/CH-SeNPs nano-composite (10 mg) was put on a platinum pan, then heated from 25°C to 500 ºC at the rate of 10 ^o^C/min in a nitrogen flow of 30–60 mL/min. PVA/CH-SeNPs nano-composite film weight loss was calculated as a function of temperature [21].

**Antioxidant activity of SeNPs, PVA/CH and PVA/CH-SeNPs nano-composite films**

The DPPH (1,1-diphenyl-2-picrylhydrazyl) radical scavenging ability of SeNPs, PVA/CH and PVA/CH-SeNPs nano-composite films was assessed as described by [22–24]. For the DPPH method, 100 μL of a freshly prepared DPPH solution (0.2 μM in methanol) was mixed with 100 μL of carotenoids (1.5–50 mg/100 mL) and left in the dark for 30 min at 24.6 ºC. The mixture's absorbance was then recorded at 517 nm. The scavenging ability of the DPPH (1,1-diphenyl-2-picrylhydrazyl) radical (% inhibition) was calculated via the following equation:

$$\% inhibition= \frac{Abs control-Abs sample}{Abs control} X 100$$

**Antimicrobial activity PVA/CH and PVA/CH-SeNPs nano-composite films**

The antibacterial activity of biosynthesized PVA/CH and PVA/CH-SeNPs nano-composite films was evaluated against five pathogenic bacterial strains: *Escherichia coli* ATCC 8379*, Klebsiella pneumoniae* ATCC 00607*, Salmonella typhi* DSM 17058*, Bacillus cereus,* and *Staphylococcus aureus*. The strains were cultivated on Mueller-Hinton Agar (MHA). The well diffusion method was used to assess the inhibitory effects of six concentrations of PVA/CH and PVA/CH-SeNPs nano-composite films. A volume of 50 μL of each bacterial inoculum (10⁶ CFU/mL) was uniformly spread on the surface of sterile MHA plates. Wells (6 mm in diameter) were created using a sterile cork borer and filled with 100 μL of PVA/Ch and PVA/CH-SeNPs nano-composite at concentrations of 1000, 500, 250, 125, 75, and 25 μg/mL. Gentamicin (100 μg/mL) was used as a positive control. The plates were incubated at 37 °C for 24 hours. The inhibition zones were measured in centimeters and recorded as inhibition zone diameters (IZD) (cm) [25] . The Activity Index (AI) was calculated according to the formula:

$$\frac{Activity index \left( AI \right)= IZD of SeNPs}{IZD of reference antibiotic}$$

**Minimum inhibitory concentration (MIC), minimum bactericidal concentration (MBC), and MIC/MBC ratio of PVA/CH-SeNPs (1%) nano-composite film.**

The MIC and MBC values were determined for the PVA/CH-SeNPs nano-composite films, which exhibited the most pronounced antimicrobial activity in preliminary diffusion assays using a two-fold serial dilution method, following Clinical and Laboratory Standards Institute (CLSI) guidelines [26]. Serial dilutions of PVA/CH-SeNPs nano-composite were prepared to obtain final concentrations of 1000 (control), 500, 250, 125, 75, 50, and 25 µg/mL. The diluted samples were added into pre-inoculated MHA plates and incubated at 37 °C for 24 hours. The MIC was defined as the lowest concentration at which no visible bacterial growth was observed. All experiments were performed in triplicate, and results are presented as mean ± standard deviation [26].

**Cytotoxicity assay of PVA/CH and PVA/CH-SeNPs nano-composite films**

The cytotoxic effects of **PVA/CH and PVA/CH-SeNPs nano-composite films** were evaluated using Vero cells (Green monkey kidney cells) obtained from Science Way Lab for Scientific Services, Cairo, Egypt, through an MTT assay. The cells were cultured in DMEM medium supplemented with 10% heat-inactivated fetal bovine serum, 100 units/mL penicillin, and 100 mg/mL streptomycin, and maintained at 37 °C in a humidified incubator with 5% CO₂. To determine the potential cytotoxicity of the films, the SRB assay was performed to assess cell viability. A 100 μL cell suspension (5 × 10³ cells) was seeded into 96-well plates and incubated in complete medium for 24 hours. The cells were then treated with 100 μL of medium containing different drug concentrations. After film exposure, cells were fixed by incubating at 4 °C for 1 hour, followed by the addition of 150 μL of 10% TCA. The TCA solution was removed, and the cells were washed five times with deionized water. Subsequently, 70 μL of SRB solution (0.4% w/v) was added, and the mixture was incubated for 10 minutes at room temperature in the dark. The plates were then air-dried overnight after three washes with 1% ethanolic acid. Finally, the SRB-stained proteins were dissolved using 150 μL of TRIS buffer (10 mM), and the absorbance was recorded at 540 nm using an Infinite F50 microplate reader (TECAN, Switzerland) [27].

**Analysis of plum fruits quality**

Total soluble solids (TSS) were quantified using a hand refractometer (HR110), with findings shown as a percentage (%) of the juice [28]. Titratable acidity (TA) was assessed by titrating 10 mL of juice to pH 8 with 0.1 N sodium hydroxide, using 1% phenolphthalein as an indicator, following AOAC (2006) standards [28]. Fruit firmness was assessed using a fruit texture analyzer (GS-15, serial No. FTA2, UP Umweltanalytische Produkte GmbH) and quantified in Newtons (N). Weight loss during storage was evaluated by weighing plum fruits using an analytical. Weight loss (%) was determined using the accompanying formula [29].

Three replicates were used for the measurements. The following formula was used to determine weight loss:

Weight loss (%) = *[(initial mass−final mass)/initial mass] x 100%*

**Anthocyanin Content**

Anthocyanin content was measured following [30]. A 0.1 g plum sample was extracted in 10 mL acidified methanol, infused for 24 h, centrifuged at 4000 × g for 10 minutes, and diluted to 10 mL. The extract was mixed with KCl buffer (pH 1.0, 25 mM) and sodium acetate buffer (pH 4.5, 0.4 M). Absorbance was recorded at 510 nm and 700 nm after 15 minutes of incubation [31]. Anthocyanin content in plum fruits was calculated as mg cyanidin-3-glucoside equivalent/100 g FW by the following equation.

$$Anthocyanin content= \frac{A \times MW\times DF \times1000}{\varepsilon\times Molar}$$

where A: Absorbance; MW: Molecular weight (449.2 g/mol); DF: Dilution Factor; ε: Molar extinction coefficient of cyanidin-3-glucoside (26,900 L Mol^-1^.cm^-1^).

**Statistical analysis**

Result data were analyzed as a two-way analysis with a probability level of 5% using SPSS for Windows. Duncan’s test (P ≤ 0.05) was exploited to differentiate between the means of treatments.

**Results**

**Quantitative profile of phenolic compounds and vitamin C in orange peel extract via HPLC analysis.**

The chromatographic analysis in **Table 1** **and Figure 2** present data on 18 phenolic compounds, including flavonoids and phenolic acids. The table shows each compound's expected and actual retention times, peak areas, and concentrations in mg/kg. Actual retention times range from 2.99 min for Gallic acid to 19.41 min for Kaempferol, with other notable compounds eluting at various times in between: Catechol (4.09 min), Chlorogenic acid (7.12 min), Rutin (11.04 min), Quercetin (17.97 min), and Apigenin (18.84 min). Retention times closely match expectations, indicating good chromatographic separation. Concentrations vary widely, from 1.79 mg/kg (Myricetin) to 546.46 mg/kg (Rutin). Notable high-concentration compounds include Rutin (546.459 mg/kg), Quercetin (217.449 mg/kg), and Chlorogenic acid (72.753 mg/kg), while compounds like Myricetin, Hesperidin, and Resveratrol are present in much lower amounts (1.790, 2.283, and 2.700 mg/kg respectively). This analysis provides a comprehensive profile of phenolic compounds in the sample, likely from a plant extract or food product. **Table 1 and Figure 2** show the HPLC analysis for ascorbic acid (vitamin C). The expected retention time was 2.200 min, which matched exactly with the observed retention time of 2.20 min. The peak area for ascorbic acid was measured at 437.513. The concentration of ascorbic acid in orange peel extract (OPE) was determined to be 0.094 mg/kg.

**Table 1:** Quantitative profile of phenolic compounds and vitamin C in orange peel extract via HPLC analysis

| **No.** | **Name** | **RT [min]** | **Area** | **Amount [mg/kg]** |
| --- | --- | --- | --- | --- |
| **Phenolic substances** | | | | |
| 1 | **Gallic acid** | 2.99 | 36.597 | 3.637 |
| 2 | **Catechol** | 4.09 | 39.735 | 13.085 |
| 3 | ***P*-hydroxybenzoic** | 5.85 | 88.369 | 22.508 |
| 4 | **Catechin** | 6.80 | 39.103 | 18.994 |
| 5 | **Chlorogenic acid** | 7.12 | 232.866 | 72.753 |
| 6 | **Vanillic acid** | 7.31 | 26.467 | 4.394 |
| 7 | **Syringic acid** | 7.91 | 42.797 | 4.077 |
| 8 | **P Coumaric** | 9.69 | 431.171 | 31.931 |
| 9 | **Rutin** | 11.04 | 3795.12 | 546.459 |
| 10 | **Ferulic** | 11.48 | 28.881 | 10.938 |
| 11 | **O-Cumaric** | 12.71 | 211.838 | 12.468 |
| 12 | **Hesperidin** | 13.85 | 11.305 | 2.283 |
| 13 | **Resveratrol** | 15.62 | 9.305 | 2.700 |
| 14 | **Rosemarinic acid** | 15.87 | 16.988 | 7.216 |
| 15 | **Myricetin** | 16.81 | 15.000 | 1.790 |
| 16 | **Quercetin** | 17.97 | 204.072 | 217.449 |
| 17 | **Apigenin** | 18.84 | 498.165 | 7.469 |
| 18 | **Kaempferol** | 19.41 | 146.116 | 20.421 |
| **Ascorbic acid** | | | | |
|  | **Ascorbic acid** | 2.20 | 437.513 | 0.094 |

**Figure 2:** HPLC chromatogram of **(a)** phenolic substances and **(b)** ascorbic acid in orange peel extract (OPE)

**Preparation of PVA/CH and PVA/CH-SeNPs nano-composite films**

**Figure 3** demonstrates the preparation of PVA/CH and PVA/CH-SeNPs at varying concentrations, four eppendorf tube and corresponding petri dishes containing solutions ranging from 0.25 to 1 %. A clear concentration-dependent color gradient is visible, progressing from light pink (0.25 % of SeNPs) to red (1% of SeNPs, indicating increasing formation of selenium nanoparticles. Part (A) displays a clear, displays the PVA/CH film. Part (B) presents concentrated form of the PVA/CH-SeNPs film.

**Figure 3:** Preparation of PVA/CH-SeNPs at varying concentrations. **A)** displays the PVA/CH film. **B)** concentrated form of the PVA/CH-SeNPs film.

**Characterization of SeNPs, PVA/CH, and PVA/CH-SeNPs composites films**

FTIR spectroscopy was employed to assess the functional groups implicated in the interaction between the carriers and bioactive substances. The FTIR spectra of SeNPs, PVA/CH, and PVA/CH-SeNPs composites are presented in **Figure 4**. The absorption peaks for the PVA/CH/SeNPs composite were found at 3423 cm⁻¹ for the broad signal of the (-OH) group and at 1617 cm⁻¹ for the protein amide I band, which is related to C–O stretching. While the absorption peaks for the PVA/CH composite are located at 3411 and 1640 cm⁻¹. Moreover, in the spectrum of PVA/CH/SeNPs, the absorption peaks at the OH group were red-shifted to 3423 cm⁻¹, indicating the new H-bond formation between SeNPs and the PVA/CH film. These results demonstrated a noticeable change in peak intensities and slight red shifts, suggesting interactions between the nanoparticles and the polymer matrix. The UV-Vis spectra in Figure 5 shows distinct absorption patterns across different samples. The UV spectra of SeNPs showed two distinct peaks at 258 and 325 nm **(see Figure 4)**. The PVA/CH spectrum shows no absorption peak, as previously reported [19]. After loading SeNPs, the two distinct peaks were red shifted to 284 and 335 nm in the PVA/CH-SeNPs spectra. This indicated the interaction formation between SeNPs and PVA/CH composite. Moreover, the peak intensity PVA/CH-SeNPs spectra decreased compared to PVA/CH spectra, proving the interaction among ligands. Similar phenomena have been observed in the study of [32]. This study demonstrated that the changes or disappearance of absorption peaks indicate that the ligand is well entrapped in biofilm formation.

**Figure 4:** Characterization of SeNPs, PVA/CH, and PVA/CH-SeNPs PVA/CH-SeNPs composites, **(a)** FTIR spectral analysis, and **(b)** UV-Vis Spectral Analysis.

**Figurers 5** **and 6** show the dynamic light scattering (DLS) and zeta potential measurements provide insights into the size distribution and stability of the nanoparticles across different samples. The present study used DLS analysis to determine the average hydrodynamic diameter of SeNPs, PVA/CH, and PVA/CH-SeNPs composites. The present study used dynamic light scattering (DLS) analysis to determine the average hydrodynamic diameter of SeNPs synthesized using an extract derived from orange peels. In this context, the hydrodynamic diameter was ascertained to be 756.1 nm **(Figure 5)**, suggesting a higher magnitude when compared to observations obtained using TEM technique. The inclusion of the selenium nanoparticle size estimate, along with the DLS analysis's consideration of capping biomolecules and the surrounding hydrate layers, may be the cause of the observed discrepancy [33]. The average hydrodynamic diameter of PVA/CH film show smaller size distributions (13.49 nm), while the PVA/CH-SeNPs composite film (0.25, 0.5 and 1%) exhibit slight variations in peak size and distribution width, possibly due to the incorporation of selenium nanoparticles.  The average hydrodynamic diameter of PVA/CH-SeNPs (0.25%) film show 672.3 nm, while average hydrodynamic diameter to 748.9 nm for PVA/CH-SeNPs (1%).

**Figure 5:** Size distribution of SeNPs, PVA/CH and PVA/CH/Se-NPs PVA/CH-SeNPs composites at varying concentrations. **A**: SeNPs; **B**: PVA/CH; **C-E:** DLS size distribution for PVA/CH-SeNPs, 0.25, 0.5, and 1% composites film respectively.

Moreover, the zeta potential value of the green synthesized SeNPs was detected to be −14.5 mV **(Figure 6)**. The surface negative charge of SeNPs might be allotted to the extract biomolecules. The zeta potential value of SeNPs increased with the addition of CH/PVA. The CH/PVA coated SeNPs shows zeta potential values of 47.4 ± 9.66, 53.6 ± 8.18 mV and 45.7 ± 6.80 for PVA/CH-SeNPs (0.25%) composite, PVA/CH-SeNPs (0.5%) composite and PVA/CH-SeNPs (1%) composite, respectively.

**Figure 6:** Zeta potential of SeNPs, PVA/CH and PVA/CH-SeNPs at varying Concentrations. **A**: SeNPs; **B**: PVA/CH; **C-E**: Zeta Potential for PVA/CH-SeNPs, 0.25, 0.5, and 1% composites respectively.

Transmission electron microscopy (TEM) technique **(Figure 7)** revealed distinct morphological characteristics of selenium nanoparticles (SeNPs) and PVA/CH and PVA/CH-SeNPs composites. The selenium nanoparticles (SeNPs) exhibited a spherical morphology, with an average particle size of 81.3 nm **(Figure 7)**. SeNPs exhibited uniform spherical morphology with clear particle boundaries, while polymeric matrices displayed varying degrees of particle distribution and agglomeration. The PVA/CH base composition showed large, irregular structures with significant clustering, with an average particle size of 37.08 nm and increasing polymer concentrations progressively modified nanoparticle dispersion and aggregation patterns. In the current study, an increase in the diameter of the CS-coated SeNPs was observed due to the presence of the CH layer on the Se surface. The mean diameter of PVA/CH-SeNPs composites film at 0.25, 0.5 and 1% was in the range 92.65, 94.25, and 90.65 nm, respectively.

**Figure 7**: Transmission Electron Microscopy (TEM) images showing morphological variations, particle size distribution, and agglomeration patterns across different compositions and concentrations.

**Stability of PVA/CH/Se-NPs composites.**

It is essential to assess the impact of several environmental factors on the stability of nanoparticles into coating films. The effect of pH (3,7 and 12) on the particle size of selenium nanoparticles into PVA/CH-SeNPs film was evaluated **(Figure 8).** The selenium nanoparticles into PVA/CH-SeNPs film showed good stability at pH level of 3, suggesting that the ionic gelation method synthesized stable nanoparticles.

**Figure 8:** Effect of pH 3 **(a),** 7 **(b)** and 12 **(c)** on PVA/CH/Se-NPs composites.

**Figure 9a** illustrates the impact of UV light duration on the PVA and CH retention rate of the synthesized selenium nanoparticles. As the exposure time increased, the PVA and CH retention rate of the selenium nanoparticles was slightly increased. When the exposure time reached 4 h, the PVA/CH-SeNPs film retention rates were 86.24 ± 3.28 %, respectively. This suggests that the PVA/CH, acting as transport carriers for Se nanoparticles, effectively shielded the SeNPs from UV light. We result showing that PVA/CH-SeNPs film was more resistant to UV decomposition. This may be due to the effectiveness of selenium as an antioxidant [34]. Encapsulation or coatings of chitosan on the surface of selenium might improve its bioavailability and carrier stability for effective drug administration. Chitosan is a natural polymer formed from chitin, characterized by its non-toxicity, biocompatibility, and biodegradability. It stabilizes nanoparticles due to its polycationic characteristics and enhances their stability [35]. The negative charges of Chitosan generate static repulsive forces on the material's surface, resulting in a stable suspension [36].

**Figure 9:** Effect of the UV light **(a)** and storage time **(b)** on the SeNPs retention rate (%) of the prepared PVA/CH-SeNPs. **(c)** The thermal degradation of PVA/CH and PVA/CH-SeNPs films**.**

**Figure 9b** illustrates the impact of storage time on retention rate of selenium nanoparticles into PVA/CH-SeNPs film. As the storage time increased, SeNPs into PVA/CH-SeNPs film retention rate significantly decreased, showing a reduction in their stability. The retention rates of SeNPs from PVA/CH-SeNPs film were 76.46 ± 1.94% after storage 16 days. The stability of nanoparticles is attributed to the electrostatic repulsion between Se nanoparticles and CH. Previous study reported that the high stability may be attributed to the CH coating layer, which acts as a barrier that protects Se from degradation, thereby inhibiting its release [19]. The DSC analysis method is employed to verify the incorporation of an active component inside a biofilm. This analysis demonstrated the potential interactions between the wall and the core, the displacement or removal of fusion peaks, and crystallisation or other thermal transformations. Therefore, **Figure 9c** displays the thermal degradation of PVA/CH and PVA/CH-SeNPs. The thermograms of the peaks detected at 103.37 and 110.102 ℃ were attributed to the moisture evaporation point for each sample as previously reported [32]. The temperature degradation of PVA/CH displayed three thermal peaks located at 244.4, 297.18, and 429.14 ℃, which was attributed to the melting point of PVA/CH. The temperature degradation of PVA/CH-SeNPs displayed two thermal peaks appearing at 288.17 and 423.07 ℃, which was attributed to the melting point of PVA/CH-SeNPs. Furthermore, the change in thermal behaviors of PVA/CH film could be attributed to the addition of SeNPs into the film matrix. This finding indicated the interaction formation between PVA/CH film and SeNPs.

**Antioxidant activity of SeNPs, PVA/CH and PVA/CH-SeNPs composite films.**

**Figure 10** presents IC50 values (µg/mL) for different treatments, including ascorbic acid, SeNPs, PVA/CH, and PVA/CH-SeNPs at varying concentrations (0.25%, 0.5%, and 1% of SeNPs). The IC50 values are displayed on a logarithmic scale. Ascorbic acid and SeNPs exhibit the lowest IC50 values. The trend indicates that incorporating SeNPs into the PVA/CH matrix reduces the IC50 value as the concentration of SeNPs increases. The PVA/CH-SeNPs (1%) film exhibited strong antioxidant activity (IC50 value 65.44 ± 2.03 µg/mL) compared to PVA/CH, and PVA/CH-SeNPs (0.25 and 0.5%) films, respectively.

**Figure 10:** IC50 values (µg/mL) of different treatments, including ascorbic acid, SeNPs, PVA/CH, and PVA/CH-SeNPs at Varying Concentrations (0.25%, 0.5%, and 1% of SeNPs). Different letters indicate statistically significant differences between treatments (p < 0.05).

**Antimicrobial efficacy of PVA/CH and PVA/CH-SeNPs composite films against pathogenic microorganisms**

**Figure 11** demonstrates the antimicrobial efficacy of PVA/CH and PVA/CH-SeNPs composite films was assessed against five pathogenic strains using agar well diffusion assays. The pristine PVA/CH film exhibited no measurable inhibition against any tested strains. In contrast, films embedded with PVA/CH-SeNPs composite film (1% SeNPs) demonstrated the highest antimicrobial activity, with inhibition zone diameters (IZD) of 2.2 ± 0.11 cm for *B. cereus*, 2.5 ± 0.125 cm for *S. aureus*, 1.5 ± 0.075 cm for *E. coli*, 0.75 ± 0.0375 cm for *K. pneumoniae*. Film containing 0.5% SeNPs showed moderately reduced activity, yielding IZDs of 1.8 ± 0.09 cm, 2.1 ± 0.105 cm, 0.9 ± 0.045 cm, 0.5 ± 0.025 cm, and 1.2 ± 0.06 cm, respectively, for the same organisms. The lowest tested PVA/CH-SeNPs (0.25%) film resulted in further diminished zones: 1.5 ± 0.075 cm for B. cereus, 1.9 ± 0.095 cm for S. aureus, 0.5 ± 0.025 cm for *E. coli*, 0.5 ± 0.025 cm for *K. pneumoniae*, and 0.9 ± 0.045 cm for *S. typhi*. These results confirm a concentration-dependent enhancement in antimicrobial activity, supporting the potential use of SeNPs-loaded PVA/Ch films in biomedical or antimicrobial packaging applications.

**Figure 11.** Inhibition zone diameters (IZD) of different PVA/CH composite films containing varying concentrations of SeNPs (0.25%, 0.5%, and 1%) against five pathogenic microbial strains: *E. coli, K. pneumoniae, S. typhi, B. cereus,* and *S. aureus*. Visual representation of antimicrobial activity on agar plates, with labeled samples: **A)** PVA/CH-SeNPs (1%), **B)** PVA/CH-SeNPs (0.5%), **C)** PVA/CH-SeNPs (0.25%), and **unlabelled** = control PVA/CH.

**Minimum inhibitory and bactericidal concentrations of PVA/CH-SeNPs (1%) composite film Against pathogenic microorganisms**

The antimicrobial efficacy PVA/CH-SeNPs (1%) composite film was further evaluated using broth microdilution assays to determine the minimum inhibitory concentration (MIC) and minimum bactericidal concentration (MBC) against five pathogenic strains. The film exhibited strong bactericidal activity against *B. cereus* and *S. aureus*, with MIC and MBC values of 250 ± 12.5 µg/mL and 500 ± 25 µg/mL, respectively, yielding a MIC/MBC ratio of 0.5. Similar bactericidal effects were observed against *E. coli* (MIC: 500 ± 25 µg/mL; MBC: 1000 ± 50 µg/mL). In contrast, *K. pneumoniae* exhibited weak susceptibility, with a high MIC of 1000 ± 50 µg/mL and no bactericidal effect observed within the tested concentration range (MBC > 1000 µg/mL), indicating a MIC/MBC ratio ≥1. These findings highlight the concentration-dependent and strain-specific antimicrobial potential of SeNPs incorporated PVA/CH films, particularly effective against Gram-positive bacterial and fungal strains.

**Table 2**: Minimum inhibitory concentration (MIC), minimum bactericidal concentration (MBC), and MIC/MBC ratio of PVA/CH-SeNPs (1%) composite film against selected pathogenic strains.

| Microorganism | MIC (µg/mL) ± SD | MBC (µg/mL) ± SD | MIC/MBC Ratio | Interpretation |
| --- | --- | --- | --- | --- |
| *Bacillus cereus* | 250 ± 12.5^a^ | 500 ± 25^a^ | 0.5 | Bactericidal |
| *Staphylococcus aureus* | 250 ± 12.5 ^a^ | 500 ± 25^a^ | 0.5 | Bactericidal |
| *Escherichia coli* | 500 ± 25 ^b^ | 1000 ± 50^b^ | 0.5 | Bactericidal |
| *Klebsiella pneumoniae* | 1000 ± 50^c^ | >1000^c^ | ≥1.0 | Weakly Inhibitory |
| *Salmonella typhi* | 250 ± 12.5^b^ | 500 ± 25^b^ | 0.5 | Fungicidal |

Values are expressed as mean ± standard deviation (SD) based on triplicate experiments. A MIC/MBC ratio ≤0.5 indicates bactericidal or fungicidal activity, while a ratio ≥1.0 suggests weak inhibitory effects. Different lowercase letters (a, b, c, within the same column indicate statistically significant differences (p < 0.05) as determined by one-way ANOVA followed by Tukey’s post hoc test.

**Cytotoxicity of PVA/CH and PVA/CH-SeNPs composite films.**

Currently, selenium nanoparticles represent a promising option in chemotherapy research due to their remarkable anticancer efficacy and minimal toxicity [37-39]. **Figure 12** shows cytotoxicity test results against Vero cell line for different substances at various concentrations. The substances tested are PVA/CH, and two concentrations of PVA/CH-SeNPs composite films at 0.25% and 0.5%. For most substances, cell viability remains high (80-100%) across all tested concentrations, except for PVA/CH-SeNPs composite films at 1% at 1000 μg/mL, where viability drops to around 50%. The results obtained showed that the cell viability reduced as the concentration of SeNPs increased. The IC_50_ value of PVA/CH-SeNPs (0.25%) composite film was about 926.89 µg/mL, and PVA/CH-SeNPs (0.5%) composite film, the IC_50_ value reached 819.57 µg/mL. PVA/CH-SeNPs (1%) composite film were tested for in vitro cytotoxicity against Vero cell lines the IC_50_ value decreased to 308.77 µg/mL.

**Figure 12:** Cytotoxicity evaluation of various substances on Vero cell line. a) IC50 of PVA/CH and PVA/CH-SeNPs composite films. **b-e)** Vero cell line morphology images of **(b)** PVA/CH, **(c)** PVA/CH-SeNPs (0.25%) composite film, **(d)** PVA/CH-SeNPs (0. 5%) composite film, **(e)** PVA/CH-SeNPs (1%) composite film.

**Fruit physical and chemical characteristics measurements**

As shown in **Figures 13, 14** and **15** the incorporation of nano-selenium enhanced the efficiency of chitosan as an edible coating, improving the cold storage period and physical and chemical quality of plum fruits during storage. While weight loss generally increased over time, the nano-selenium treatments significantly reduced it compared to the control. The lowest weight loss was observed in PVA/CH-SeNPs at 0.5% (3.64%) and 1% (2.84%), with no significant difference between them up to 21 days. After 28 days, weight loss remained lowest in the PVA/CH-SeNPs 1% treatment.

**Figure 13:** Impact of PVA/CH and PVA/CH-SeNPs films on enhancing storability of Hollywood plum during cold storage conditions (at 0±2°C and 90 ±5% RH).

The treatments also maintained fruit firmness, preventing excessive softening over storage. After 28 days, fruits coated with PVA/CH-SeNPs at 1% had a firmness of 7.198 N/cm², compared to 4.12 N/cm² in the control. Regarding chemical quality, the nano-selenium coatings led to a higher total soluble solids (TSS) content and a lower acidity percentage. Additionally, the coatings significantly increased L-ascorbic acid and anthocyanin content, preserving the fruit's nutritional quality. The best results were observed with PVA/CH-SeNPs at 0.5% and 1%, with no significant differences between them (TSS: 14.4 and 14.6, titratable acidity: 0.936, TSS/TA ratio: 15.38 and 15.59, anthocyanin: 19.78 and 19.86 mg/100 mg of fruit, respectively).Top of Form

Bottom of Form

**Figure 14**. Impact of PVA/CH and PVA/CH-SeNPs films on physical characteristics of Hollywood plum fruits during cold storage conditions (at 0±2°C and 90 ±5% RH): (**a)** weight loss %, (**b)** firmness (N/cm^2^).

**Figure 15.** Impact of PVA/CH and PVA/CH-SeNPs films on chemical characteristics of Hollywood plum fruits during cold storage conditions (at 0±2°C and 90 ±5% RH): (**a)** total soluble solids (°Brix) (**b)** titratable acidity (%) (**c)** TSS/acidity ratio (**d)** L-Ascorbic acid (mg/100 mg of fruit) (**e)** Anthocyanin (mg/100 mg of fruit).

**Discussion**

The data presented in this study provide significant insights into the synthesis, characterization, and biological activity of SeNPs incorporated into a PVA/Ch matrix. The antimicrobial, antioxidant, and cytotoxic properties of the SeNP composites were evaluated, and the results indicate their potential for use in biomedical applications. In this section, we will discuss the possible explanations for the observed results, drawing comparisons with findings from other studies [40].

The successful synthesis of SeNPs was confirmed through UV-Vis spectroscopy, FTIR, DLS, zeta potential analysis, and size distribution data. The UV-Vis spectra revealed a strong absorption peak around 258-325 nm, which is characteristic of selenium nanoparticles. This observation aligns with previous studies, where SeNPs typically exhibit similar absorption profiles, confirming the formation of NPs with optical properties specific to selenium [41]. The PVA/CH spectrum shows no absorption peak, as previously reported [19]. After loading SeNPs, the two distinct peaks were red shifted to 284 and 335 nm in the PVA/CH-SeNPs spectra.

The FTIR spectra showed distinctive peaks corresponding to functional groups in the PVA/Ch-SeNPs composite, including O-H, C-H, and C-O stretching vibrations. These peaks suggest strong interactions between SeNPs and the PVA/Ch matrix, likely responsible for stabilizing the nanoparticles within the polymer matrix. These functional groups are consistent with other studies where chitosan and PVA have been used to stabilize selenium nanoparticles, allowing for a more controlled and uniform nanoparticle distribution.

DLS and zeta potential analyses provided further insights into the synthesized nanoparticles' size distribution and surface charge. The average particle size of the SeNPs was approximately 81.3 nm. with a narrow distribution within the range of previously reported SeNPs. The PVA/CH base composition showed large, irregular structures with significant clustering, with an average particle size of 37.08 nm and increasing polymer concentrations progressively modified nanoparticle dispersion and aggregation patterns. In the current study, an increase in the diameter of the CS-coated SeNPs was observed due to the presence of the CH layer on the Se surface. The mean diameter of PVA/CH-SeNPs composites film at 0.25, 0.5 and 1% was in the range 92.65, 94.25, and 90.65 nm, respectively. This is in agreement with [35] findings.

The negative zeta potential values (-14.5 mV) suggest good colloidal stability, preventing nanoparticle aggregation. Similar studies have shown that SeNPs with zeta potential values below -20 mV generally exhibit high stability due to electrostatic repulsion between particles. This stability is crucial for ensuring the long-term effectiveness of the SeNP composites, particularly in biomedical applications where stability can influence biological interactions and efficacy [42]. The composite samples (PVA/CH and PVA/CH/Se-NPs) show some variation in zeta potential compared to selenium nanoparticles, suggesting changes in surface properties with the addition of selenium nanoparticles and chitosan. PVA/CH-SeNPs composites exhibited positive zeta potential values. Similarly, Gosala et al. 2025 [35] reported that the CS-Se@DNR revealed positive zeta potential values. The presence of chitosan (CH) on the Se surface may contribute to its stability. Positive zeta potential values were obtained, indicating acceptable stability. The change in zeta potential values indicates a positively charged exterior for the nanocarrier [43]. The presence of NH3^+^ groups on the CS may be the source of the positive charge.

The stability of PVA/CH loaded with selenium nanoparticles (SeNPs) is affected by several factors, such as the concentration of SeNPs, the molecular weight of chitosan, and environmental conditions. Studies demonstrate that the integration of SeNPs improves the antibacterial efficacy of the PVA/CH matrix while preserving stability under certain conditions. Increased concentrations of SeNPs correlated with improved antibacterial effectiveness, evidenced by the larger inhibition zones reported in laboratory tests [44].. The optical and structural properties of the PVA/CH films change with varying SeNP concentrations, indicating a significant interaction between the constituent materials [44].

A comprehensive investigation was undertaken to examine the impact of technological parameters on the stability of selenium nanoparticles stabilized via chitosan. The findings revealed that an augmentation in exposure duration correlates with an increase in the mean hydrodynamic radius of chitosan-stabilized selenium nanoparticles [45]. Conversely, in relation to pH, an inverse correlation is evident: the samples exhibiting the largest mean hydrodynamic radius are those situated within an acidic milieu (pH < 5). Within the framework of the analysis concerning the effect of technological parameters on the stability of chitosan-stabilized selenium nanoparticles, it was determined that these nanoparticles can serve as a selenium source for food products characterized by a neutral pH, while being amenable to thermal processing at temperatures exceeding 70 °C for durations of 5 to 15 minutes, notably in pasteurized milk. An examination of pasteurized milk fortified with selenium nanoparticles has also been conducted [45].

Yu et al. [46] demonstrated that PVA-SeNPs possess significant stability in both mono- and polyvalent electrolytes due to the pronounced steric hindrance provided by the capping agent, whereas Alg-SeNPs exhibit minimal size augmentation in elevated salt concentrations but tend to assemble in specific divalent solutions. The results showed that incorporating SeNPs increased the thermal stability of the PVA/CH-SeNPs film. The interaction of SeNPs and PVA/CH results in stable nanoparticles with physical, thermal, and morphological properties, as well as activity against free radicals and pathogens.

The antioxidant activity of the PVA/CH-SeNPs composites, as indicated by their ability to scavenge free radicals, increased with higher SeNP concentrations. This result is consistent with other studies, which have demonstrated that selenium nanoparticles possess significant antioxidant properties, largely due to their ability to interact with and neutralize ROS. The incorporation of SeNPs into the PVA/CH matrix likely enhanced the stability and bioavailability of the nanoparticles, leading to more effective ROS scavenging compared to the free nanoparticles [47].

The antioxidant effect observed in this study may be attributed to the intrinsic properties of selenium. Selenium is a key component of several antioxidant enzymes, including glutathione peroxidase, critical in reducing oxidative stress in biological systems. SeNPs, due to their nanoscale size and high surface area, may further enhance these enzymatic activities, providing a more efficient mechanism for neutralizing ROS. Furthermore, using a biocompatible matrix like PVA/CH helps protect the nanoparticles from degradation, ensuring sustained antioxidant activity [48].

Previous research has shown that antioxidant activity in SeNPs is concentration-dependent, with higher concentrations leading to more effective ROS neutralization. The results from our study corroborate these findings, suggesting that the enhanced antioxidant activity observed in the higher SeNP concentration composites is likely due to increased ROS scavenging ability facilitated by the high surface area of the nanoparticles and their uniform distribution within the PVA/CH matrix [49].

The antimicrobial results demonstrated that the PVA/CH-SeNPs composites exhibited concentration-dependent antibacterial activity against a range of pathogenic bacteria. The inhibition zones for PVA/CH-SeNPs composites increased with higher nanoparticle concentration, which suggests that the nanoparticles play a key role in the observed antimicrobial effects. This trend has been corroborated by previous research, where SeNPs were shown to exert antimicrobial effects through generating ROS and disrupting bacterial cell membranes [50].

Interestingly, the PVA/CH-SeNPs composites exhibited significant antibacterial activity, particularly against Gram-positive bacteria. Chitosan, a known antimicrobial agent, likely contributed to the overall efficacy of the composite. Chitosan's antimicrobial mechanism is thought to involve the interaction of its positively charged amino groups with the negatively charged microbial cell membranes, leading to cell wall disruption and leakage of intracellular contents. This synergy between the antimicrobial properties of chitosan and the ROS-generating ability of SeNPs may explain the enhanced activity observed in the PVA/CH-SeNP composites, particularly at higher concentrations. Studies by [51-54] have demonstrated similar synergies between chitosan and nanoparticles like silver and gold in antimicrobial formulations [55].

Moreover, the observed variation in antimicrobial efficacy between different bacterial strains can be attributed to differences in cell wall structure. Gram-positive bacteria (*S. aureus*) are more susceptible to both SeNPs and chitosan, likely due to their thicker peptidoglycan layer, which is more vulnerable to the disruptive effects of ROS and cationic polymers like chitosan. In contrast, Gram-negative bacteria (*K. pneumoniae*) possess an outer membrane that offers additional protection, rendering them less susceptible to the antimicrobial effects of the SeNP composites. This finding is consistent with prior research highlighting bacterial strains' differential susceptibility based on their cell wall structures [56].

Selenium intake should be between 55-75 μg/day, with an upper limit of ∼400 μg. It can be taken from typical foods including mushrooms, vegetables, cereals, and food additives in a tea drink that claims to have various health benefits [57].Nano-selenium is more biologically active, has a higher bioavailability, and is less toxic than organic and inorganic Se molecules like Se (IV) and Se (VI) [58].

The cytotoxicity assay against Vero cells revealed that SeNPs and their composites exhibited low toxicity, with cell viability remaining above 80% at most concentrations. These results suggest that the PVA/CH-SeNPs composites are biocompatible, making them suitable for biomedical applications. This finding aligns with previous studies, which reported that SeNPs exhibit selective cytotoxicity, being more toxic to cancer cells than normal cells [59].

The low toxicity of SeNPs, particularly at moderate concentrations, may be attributed to their ability to be taken up by cells in a controlled manner. Studies have shown that SeNPs are internalized by cells through endocytosis, where they can interact with intracellular components without causing significant damage to healthy cells. Furthermore, the PVA/CH matrix likely reduces cytotoxicity by controlling the release of SeNPs, thereby preventing excessive exposure to nanoparticles [60]. However, the cytotoxicity levels observed in this study were still within acceptable limits for biomedical applications, further supporting the potential of these composites in therapeutic contexts [61].

The HPLC analysis provided a detailed phenolic profile of the OPE, which was used as a reducing and stabilizing agent in the synthesis of SeNPs. Phenolic compounds such as rutin, quercetin, and chlorogenic acid were identified in high concentrations, with rutin being the most abundant. These compounds are well-known for their antioxidant properties and may have contributed to the enhanced antioxidant activity of the PVA/CH-SeNP composites. The use of plant extracts for nanoparticle synthesis not only provides a green and eco-friendly method but also enhances the biological activity of the resulting nanoparticles due to the presence of bioactive compounds. [62,63].

Additionally, there were no other morphologies seen, and the measured morphologies of the chemically produced
The nanostructure was obviously made up of nanoparticles. Depending on the reductive chemicals used in the nanoparticle synthesis process, different selenium nanoparticle morphologies were produced [64]. El-Zayat et al. [64] claim that the synthesis of selenium nanoparticles using an extract of *Ephedra aphylla* stems demonstrated that the chemical constituents phenolic, flavonoid, tannin, and alkaloids were present and were responsible for the stability and biosynthesis of the nanoparticles, resulting in higher stability and a range of desired morphologies.

Edible coating enhances the freshness of fruit while preserving its nutritional content. These coatings not only extend shelf life but also promote environmental sustainability by reducing the need for synthetic preservatives. Coating treatments significantly reduced weight loss in plum fruits by preserving moisture on their surface. Desiccation in fruit tissues results in physiological alterations, degradation of quality, and diminished market value. Modified coating processes and edible coatings considerably decrease water loss by establishing a protective barrier, so preserving texture, appearance, and commercial quality throughout storage [65]. The reduction in weight loss in chitosan-treated fruits may be attributed to the formation of a high relative humidity atmosphere around the fruits, which reduces water vapor transmission and respiration rates [66]. Edible coatings function as a barrier, inhibiting water vapor migration from the fruit and decreasing transpiration and moisture loss. These coatings also modulate gas exchange, governing the transference of water vapor, oxygen, and carbon dioxide, so sustaining ideal humidity levels, and averting severe dehydration. A progressive loss in fruit firmness was seen over the storage period. Chitosan-based coatings effectively kept firmness at 78% and reduced weight loss by around 52% compared to uncoated apples. This suggests that chitosan coatings' structural integrity and moisture retention contribute to prolonged freshness and quality during storage [67].

Chitosan coatings preserve fruit firmness by altering the atmospheric conditions around the fruit surface, inhibiting the degradation of insoluble protopectin into pectic acid and pectin [68]. Furthermore, they provide an environment characterized by elevated CO₂ levels and diminished O₂ levels, which inhibits the action of cell-softening enzymes and decelerates the breakdown of structural components like pectin and protopectin, thereby maintaining firmness during storage [68]. Chitosan, a natural biopolymer with antibacterial characteristics, enhances fruit freshness and increases shelf life, significantly influencing organoleptic aspects [69,70]. During postharvest storage, total soluble solids (TSS) briefly rise owing to the hydrolysis of insoluble polysaccharides into sugars, hence increasing sucrose content. The titratable acidity (TA) of control samples decreased significantly during storage in contrast to chitosan-coated fresh-cut kiwifruit, indicating that the coating reduced metabolic activity and inhibited acid breakdown, hence preserving elevated acidity levels postharvest [71]. This was advantageous for storage, since elevated TA coincides with reduced pH, which aids in inhibiting microbial proliferation. The LBL chitosan-alginate covering enhanced fruit preservation by modulating metabolic responses and decelerating the respiration process [72]. Ascorbic acid (vitamin C) serves a vital function as an antioxidant, eliminating reactive oxygen species (ROS) from fruits. In covered plums, ascorbic acid concentrations remained consistent throughout storage, similar to observations in fresh-cut pineapple [15]. This indicates that the coatings restricted oxygen permeability and inhibited enzyme activity, hence reducing ascorbic acid oxidation. The efficacy of edible coatings in maintaining total phenolic content is contingent upon the coating ingredients and the kind of fruit. In contrast to the separate applications of sodium alginate or chitosan, the layer-by-layer (LBL) chitosan-alginate treatment demonstrated superior efficacy in diminishing respiration, resulting in elevated ascorbic acid and phenolic content, alongside reduced TSS and TA values, signifying enhanced preservation of fruit quality [73]. The color of fruit is a fundamental factor for grading by both growers and consumers, affecting purchasing choices [74]. The hue angle is a dependable metric indicating the color shift from green to purple during plum ripening [75]. The reduction in hue angle during storage is attributable to postharvest metabolism and the buildup of anthocyanins. The delay in anthocyanin accumulation in LBL-coated fruits was less than in the control, likely attributable to the suppression of respiration and anthocyanin-associated enzymes. Anthocyanins, part of the flavonoid family, provide purple, blue, orange, and red coloration to fruits, shield plants from UV radiation, and mitigate ROS [76–78]

Pear fruits subjected to selenium treatment preserved their firmness, total soluble solids, and sugar-to-acid ratio. Selenium has shown efficacy in reducing ethylene production, thereby enhancing the quality and shelf life of fruits. Nano-selenium helps maintain fruit quality by reducing weight loss and preserving firmness. Research indicates that applying nano-selenium to strawberries might markedly decrease weight loss and preserve firmness for extended durations relative to untreated fruits [51]. Selenium therapy delayed ripening and prolonged the shelf life of tomato fruits by decreasing ethylene production and augmenting the antioxidant defense system [78].

**Conclusions**

This study demonstrated the successful eco-friendly synthesis of selenium nanoparticles (SeNPs) using orange peel extract and their incorporation into a polyvinyl alcohol/chitosan (PVA/CH) composite. Characterization confirmed the formation of stable SeNPs with strong antimicrobial, antioxidant, and biocompatible properties. The antimicrobial evaluation revealed that PVA/CH-SeNPs exhibited concentration-dependent antibacterial activity, with the highest inhibition observed at 1% SeNP concentration, particularly against *S. aureus* and *S. typhi*. Antioxidant assays confirmed enhanced free radical scavenging activity, with IC50 values close to ascorbic acid, demonstrating their potential as effective antioxidant agents. Cytotoxicity tests showed that the composites maintained high cell viability (>80%), indicating their biocompatibility for biomedical applications. In addition to their biomedical potential, PVA/CH-SeNP composites were tested as an edible coating for Hollywood plum fruits, effectively extending postharvest storage life. The coatings reduced weight loss, maintained fruit firmness, and enhanced biochemical properties, including higher total soluble solids, increased L-ascorbic acid, and anthocyanin content, with 0.5% and 1% SeNP treatments providing the best results. Overall, these findings highlight the multifunctional potential of PVA/CH-SeNP composites as antimicrobial, antioxidant, and food preservation agents.

**Top of Form**

**Bottom of Form**

**References**

[1]	Ferro C, Florindo HF, Santos HA. Selenium Nanoparticles for Biomedical Applications: From Development and Characterization to Therapeutics. Adv Healthc Mater 2021;10. <https://doi.org/10.1002/adhm.202100598> .

[2]	Ahmad F, Salem-Bekhit MM, Khan F, Alshehri S, Khan A, Ghoneim MM, Wu HF, Taha EI, Elbagory I. Unique Properties of Surface-Functionalized Nanoparticles for Bio-Application: Functionalization Mechanisms and Importance in Application. Nanomaterials 2022;12. <https://doi.org/10.3390/nano12081333> .

[3]	K D, Venugopal S. Therapeutic potential of selenium nanoparticles. Frontiers in Nanotechnology 2022;4. <https://doi.org/10.3389/fnano.2022.1042338> .

[4]	Bisht N, Phalswal P, Khanna PK. Selenium nanoparticles: A review on synthesis and biomedical applications. Mater Adv 2022;3:1415–31. <https://doi.org/10.1039/d1ma00639h> .

[5]	Mikhailova EO. Selenium Nanoparticles: Green Synthesis and Biomedical Application. Molecules 2023;28. <https://doi.org/10.3390/molecules28248125> .

[6]	Chaudhary S, Mehta SK. Selenium nanomaterials: Applications in electronics, catalysis and sensors. J Nanosci Nanotechnol 2014;14:1658–74. <https://doi.org/10.1166/jnn.2014.9128> .

[7]	Zambonino MC, Quizhpe EM, Mouheb L, Rahman A, Agathos SN, Dahoumane SA. Biogenic Selenium Nanoparticles in Biomedical Sciences: Properties, Current Trends, Novel Opportunities and Emerging Challenges in Theranostic Nanomedicine. Nanomaterials 2023;13. <https://doi.org/10.3390/nano13030424> .

[8]	Zambonino MC, Quizhpe EM, Jaramillo FE, Rahman A, Vispo NS, Jeffryes C, Dahoumane SA. Green synthesis of selenium and tellurium nanoparticles: Current trends, biological properties and biomedical applications. Int J Mol Sci 2021;22:1–34. <https://doi.org/10.3390/ijms22030989> .

[9]	Ovais M, Khalil AT, Islam NU, Ahmad I, Ayaz M, Saravanan M, Shinwari ZK, Mukherjee S. Role of plant phytochemicals and microbial enzymes in biosynthesis of metallic nanoparticles. Appl Microbiol Biotechnol 2018;102:6799–814. <https://doi.org/10.1007/s00253-018-9146-7> .

[10]	Ramachandran T, Manoharan D, Natesan S, Rajaram SK, Karuppiah P, Shaik MR, Khan M, Shaik B. Synthesis and Structural Characterization of Selenium Nanoparticles-*Bacillus sp*. MKUST-01 Exopolysaccharide (SeNPs-EPS) Conjugate for Biomedical Applications. Biomedicines 2023;11. <https://doi.org/10.3390/biomedicines11092520> .

[11]	Rafique A, Mahmood Zia K, Zuber M, Tabasum S, Rehman S. Chitosan functionalized poly(vinyl alcohol) for prospects biomedical and industrial applications: A review. Int J Biol Macromol 2016;87:141–54. <https://doi.org/10.1016/j.ijbiomac.2016.02.035> .

[12]	Deng H, Su J, Zhang W, Khan A, Sani MA, Goksen G, Kashyap P, Ezati P, Rhim JW. A review of starch/polyvinyl alcohol (PVA) blend film: A potential replacement for traditional plastic-based food packaging film. Int J Biol Macromol 2024;273. <https://doi.org/10.1016/j.ijbiomac.2024.132926> .

[13]	Liu W, Golshan NH, Deng X, Hickey DJ, Zeimer K, Li H, Webster TJ. Selenium nanoparticles incorporated into titania nanotubes inhibit bacterial growth and macrophage proliferation. Nanoscale 2016;8:15783–94. <https://doi.org/10.1039/c6nr04461a> .

[14]	Omoba OS, Obafaye RO, Salawu SO, Boligon AA, Athayde ML. HPLC-DAD phenolic characterization and antioxidant activities of ripe and unripe sweet orange peels. Antioxidants 2015;4:498–512. <https://doi.org/10.3390/antiox4030498> .

[15]	Bierhals VS, Chiumarelli M, Hubinger MD. Effect of Cassava Starch Coating on Quality and Shelf Life of Fresh-Cut Pineapple (*Ananas Comosus L. Merril cv “Pérola”*). J Food Sci 2011;76. <https://doi.org/10.1111/j.1750-3841.2010.01951.x> .

[16]	Barrales FM, Silveira P, Barbosa P de PM, Ruviaro AR, Paulino BN, Pastore GM, Macedo GA, Martinez J. Recovery of phenolic compounds from citrus by-products using pressurized liquids — An application to orange peel. Food and Bioproducts Processing 2018;112:9–21. <https://doi.org/10.1016/j.fbp.2018.08.006> .

[17]	Menazea AA, Ismail AM, Awwad NS, Ibrahium HA. Physical characterization and antibacterial activity of PVA/Chitosan matrix doped by selenium nanoparticles prepared via one-pot laser ablation route. Journal of Materials Research and Technology 2020;9:9598–606. <https://doi.org/10.1016/j.jmrt.2020.06.077> .

[18]	Amr M, Abu-Hussien SH, Ismail R, Aboubakr A, Wael R, Yasser M, Hemdan B, El-Sayed SM, Bakry A, Ebeed NM, Elhariry H, Galal A, Abd-Elhalim BT. Utilization of biosynthesized silver nanoparticles from Agaricus bisporus extract for food safety application: synthesis, characterization, antimicrobial efficacy, and toxicological assessment. Sci Rep 2023;13. <https://doi.org/10.1038/s41598-023-42103-3> .

[19]	Salah M, Gong W, Tu T, Sobhy R, Dabbour M, Fang Y, Walayat N, Wang Y. Enhancing the antifungal efficiency of chitosan nanoparticle via interacting with didymin/flavonoid and its bio-based approaches for postharvest preservation in pear fruit models. Int J Biol Macromol. 2025 Apr;304(Pt 2):140889. doi:10.1016/j.ijbiomac.2025.140889.

[20]	El-Sayed, E., Hikal, M., Abo El- Khair, B., El-Ghobashy, R., & El-Assar, A. Hypoglycemic and Hypolipidimic Effects of Spirulina Platensis, Phycocyanin, Phycocyanopeptide and Phycocyanobilin on Male Diabetic Rats. 2018, Arab Universities Journal of Agricultural Sciences, 26 (Special issue (2A)), 1121-1134. doi: 10.21608/ajs.2018.28365.

[21]	Amal W. Ayoub, Sherief M. Sayed, Mahmoud A. Ammar, Yehia A. Hefnawy, Ahmed M. Youssef (2023). Novel Edible Bionanocomposites Films Based on Lemon Grass Nanoemulsion and ZnO-NPs for Extending the Shelf Life of Chilled Chicken Meat. Biointerface Research in Applied Chemistry, 13, 6, 2023, 573 https://doi.org/10.33263/BRIAC136.573

[22]	El-Sayed, S.M., Abd-Elhalim, B.T., El-Dougdoug, K.A. et al. In vitro investigation of Mangifera indica L. peel extracts: antibacterial, antioxidant, and docking studies. AMB Expr 15, 73 (2025). <https://doi.org/10.1186/s13568-025-01882-w>.

[23]	Duca A, Sturza A, Moacă EA, Negrea M, Lalescu VD, Lungeanu D, Dehelean CA, Muntean DM, Alexa E. Identification of resveratrol as bioactive compound of propolis from western Romania and characterization of phenolic profile and antioxidant activity of ethanolic extracts. Molecules 2019;24. <https://doi.org/10.3390/molecules24183368> .

[24]	Haseeb HA, Khan MA, Rasheed H, Zahid MU, Doan TD, Siddique MAR, Ahmad U, Bokhari SAI. *Polygonum bistorta* Linn. as a green source for synthesis of biocompatible selenium nanoparticles with potent antimicrobial and antioxidant properties. BioMetals 2024. <https://doi.org/10.1007/s10534-024-00622-0> .

[25]	Truong LB, Medina-Cruz D, Mostafavi E, Rabiee N. Selenium nanomaterials to combat antimicrobial resistance. Molecules 2021;26. <https://doi.org/10.3390/molecules26123611> .

[26]	Amr M, Abu-Hussien SH, Ismail R, Aboubakr A, Wael R, Yasser M, Hemdan B, El-Sayed SM, Bakry A, Ebeed NM, Elhariry H, Galal A, Abd-Elhalim BT. Utilization of biosynthesized silver nanoparticles from Agaricus bisporus extract for food safety application: synthesis, characterization, antimicrobial efficacy, and toxicological assessment. Sci Rep 2023;13. <https://doi.org/10.1038/s41598-023-42103-3> .

[27]	Abdo, R.A., Hazem, M.M., El-Assar, A.EM. et al. Efficacy of nano-silicon extracted from rice husk to modulate the physio-biochemical constituents of wheat for ameliorating drought tolerance without causing cytotoxicity. Beni-Suef Univ J Basic Appl Sci 13, 75 (2024). <https://doi.org/10.1186/s43088-024-00529-2>.

[28]	Nasser MA, El-Mogy MM, Samaan MSF, Hassan KM, El-Sayed SM, Alsubeie MS, Darwish DBE, Mahmoud SF, Al-Harbi NA, Al-Qahtani SM, Alzuaibr FM, Abd El-Gawad HG. Postharvest Exogenous Melatonin Treatment of Table Grape Berry Enhances Quality and Maintains Bioactive Compounds during Refrigerated Storage. Horticulturae 2022;8. <https://doi.org/10.3390/horticulturae8100860> .

[29]	Zhao Y, Zhu X, Hou Y, Pan Y, Shi L, Li X. Effects of harvest maturity stage on postharvest quality of winter jujube (Zizyphus jujuba Mill. cv. Dongzao) fruit during cold storage. Sci Hortic 2021;277. <https://doi.org/10.1016/j.scienta.2020.109778> .

[30]	Giusti, M.M.; Wrolstad, R.E. Characterization and Measurement of Anthocyanins by UV-Visible Spectroscopy. Curr. Protoc. Food Anal. Chem. 2001, 1042, F1.2.1–F1.2.13.

[31]	El-Beltagi HS, El-Sayed SM, Abdelhamid AN, Hassan KM, Elshalakany WA, Nossier MI, Alabdallah NM, Al-Harbi NA, Al-Qahtani SM, Darwish DBE, Abbas ZK, Ibrahim HA. Potentiating Biosynthesis of Alkaloids and Polyphenolic Substances in *Catharanthus roseus* Plant Using ĸ-Carrageenan. Molecules 2023;28. <https://doi.org/10.3390/molecules28083642> .

[32]	Karunya Saravanan, Manivannan Madhaiyan, Prabu Periyasamy, Prasath Manivannan, Alpaslan Bayrakdar, V. Balakrishnan (2025). Green synthesis and detailed characterization of selenium nanoparticles derived from Alangium salviifolium (L.f) Wangerin, Chemical Physics Impact, 10,100876, <https://doi.org/10.1016/j.chphi.2025.100876>.

[33]	Alikord, M.; Shariatifar, N.; Saraji, M.; Jahed Khaniki, G.; Hosseini, H.; Fazeli, M. Biosynthesis of Zinc Oxide Nanoparticles Using Fermented Table Olive Extract: A Novel and Green Approach with Potential Applications. BioNanoScience 2023, 13, 1036–1051.

[34]	Geoffrion, L.D., T. Hesabizadeh, D. Medina-Cruz, M. Kusper, P. Taylor, A. Vernet- Crua, J. Chen, A. Ajo, T.J. Webster, G. Guisbiers, Naked selenium nanoparticles for antibacterial and anticancer treatments, ACS Omega 5 (6) (2020) 2660–2669, <https://doi.org/10.1021/acsomega.9b03172>.

[35]	Radha Gosala, Raghunandhakumar Subramanian and Balakumar Subramanian (2025). Modulating drug delivery with nano-selenium capped by chitosan reverse micelles for anticancer potential Journal of Drug Delivery Science and Technology 108 (2025) 106860, <https://doi.org/10.1016/j.jddst.2025.106860>.

[36]	M. Collado-Gonz´alez, M.G. Montalb´an, J. Pe˜na-García, H. P´erez-S´anchez, G. Víllora, F.G. Díaz Ba˜nos, Chitosan as stabilizing agent for negatively charged nanoparticles, Carbohydr. Polym. 161 (2017) 63–70, https://doi.org/10.1016/j. carbpol.2016.12.043.

[37]	Tan H.W., Mo H.Y., Lau A.T., Xu Y.M. Selenium species: current status and potentials in cancer prevention and therapy. Int. J. Mol. Sci. 2019;20:75. doi: 10.3390/ijms20010075.

[38]	Collery P. Strategies for the development of selenium-based anticancer drugs. J. Trace Elem. Med. Biol. 2018;50:498–507. doi: 10.1016/j.jtemb.2018.02.024.

[39]	Li T., Xu H. Selenium-containing nanomaterials for Cancer treatment. Cell Rep. Phys. Sci. 2020.

[40]	Sowmya R, Karthick Raja Namasivayam S, Krithika Shree S. A Critical Review on Nano-selenium Based Materials: Synthesis, Biomedicine Applications and Biocompatibility Assessment. J Inorg Organomet Polym Mater 2024. <https://doi.org/10.1007/s10904-023-02959-4> .

[41]	Sans-Serramitjana E, Gallardo-Benavente C, Melo F, Pérez-Donoso JM, Rumpel C, Barra PJ, Durán P, Mora M de LL. A Comparative Study of the Synthesis and Characterization of Biogenic Selenium Nanoparticles by Two Contrasting *Endophytic Selenobacteria*. Microorganisms 2023;11. <https://doi.org/10.3390/microorganisms11061600> .

[42]	Zambonino MC, Quizhpe EM, Mouheb L, Rahman A, Agathos SN, Dahoumane SA. Biogenic Selenium Nanoparticles in Biomedical Sciences: Properties, Current Trends, Novel Opportunities and Emerging Challenges in Theranostic Nanomedicine. Nanomaterials 2023;13. <https://doi.org/10.3390/nano13030424> .

[43]	Lazaridou,M., E. Christodoulou, M. Nerantzaki, M. Kostoglou, D.A. Lambropoulou, A. Katsarou, K. Pantopoulos, D.N. Bikiaris, Formulation and in-vitro characterization of chitosan-nanoparticles loaded with the iron chelator deferoxamine mesylate (DFO), Pharmaceutics 12 (3) (2020) E238, https://doi.org/ 10.3390/pharmaceutics12030238.

[44]	Menazea, A. A., Ismail, A. M., Awwad, N. S., & Ibrahium, H. A. (2020). Physical characterization and antibacterial activity of PVA/Chitosan matrix doped by selenium nanoparticles prepared via one-pot laser ablation route. Journal of Materials Research and Technology, 9(5), 9598–9606. <https://doi.org/10.1016/J.JMRT.2020.06.077>.

[45]	Блинов, А. В., Gvozdenko, A. A., Blinova, A. A., Rekhman, Z., Nagdalian, A., Леонтьев, П. С., Askerova, A., & Ребезов, М. (2024). Selenium nanoparticles stabilized with chitosan for fortifying dairy products. Agrarnaâ Nauka, 1(9), 130–135. <https://doi.org/10.32634/0869-8155-2024-386-9-130-135>.

[46]	Yu, S., Liu, H., Yang, R., Zhou, W., & Liu, J. (2022). Aggregation and stability of selenium nanoparticles: Complex roles of surface coating, electrolytes and natural organic matter. Journal of Environmental Sciences-China, 130, 14–23. <https://doi.org/10.1016/j.jes.2022.10.025>.

[47]	Swarnakar NK, Jain AK, Singh RP, Godugu C, Das M, Jain S. Oral bioavailability, therapeutic efficacy and reactive oxygen species scavenging properties of coenzyme Q10-loaded polymeric nanoparticles. Biomaterials 2011;32:6860–74. <https://doi.org/10.1016/j.biomaterials.2011.05.079> .

[48]	Yang W, Owczarek JS, Fortunati E, Kozanecki M, Mazzaglia A, Balestra GM, Kenny JM, Torre L, Puglia D. Antioxidant and antibacterial lignin nanoparticles in polyvinyl alcohol/chitosan films for active packaging. Ind Crops Prod 2016;94:800–11. <https://doi.org/10.1016/j.indcrop.2016.09.061> .

[49]	Kumar A, Behl T, Chadha S. Synthesis of physically crosslinked PVA/Chitosan loaded silver nanoparticles hydrogels with tunable mechanical properties and antibacterial effects. Int J Biol Macromol 2020;149:1262–74. <https://doi.org/10.1016/j.ijbiomac.2020.02.048> .

[50]	Ahmed F, Zhang D, Tang X, Malakar PK. Targeting Spore-Forming Bacteria: A Review on the Antimicrobial Potential of Selenium Nanoparticles. Foods 2024;13. <https://doi.org/10.3390/foods13244026> .

[51]	Mahmoud TSM, Shaaban FKM, El-Hadidy GAE-M. Enhancement of antioxidant and storability of Hollywood plum cultivar by preharvest treatments with moringa leaf extract and some nutrients. Bull Natl Res Cent 2020;44. <https://doi.org/10.1186/s42269-020-00384-y> .

[52]	Duca A, Sturza A, Moacă EA, Negrea M, Lalescu VD, Lungeanu D, Dehelean CA, Muntean DM, Alexa E. Identification of resveratrol as bioactive compound of propolis from western Romania and characterization of phenolic profile and antioxidant activity of ethanolic extracts. Molecules 2019;24. <https://doi.org/10.3390/molecules24183368> .

[53]	Aziz, N. ., Alhajouj, S. A. ., Basit, A. ., Khan, I. A. ., Alaida, M. F. ., Alzayed, R. M. ., Albalawi, M. A. ., Alshareef, S. A. ., Al Duais, M. A. ., Sakran, M. ., Almalki, R. S. ., El-Khouly, A. S. ., & El Sabagh, A. . (2025). Green synthesis and antibacterial activity of silver and gold nanoparticles using crude flavonoids extracted from Bombax ceiba flowers: Bioeffects of AgNPs and AuNPs from Bombax ceiba. Cellular and Molecular Biology, 71(2), 127–135. <https://doi.org/10.14715/cmb/2025.71.2.18>.

[54]	Snoussi, M. ., Hadj Lajimi, R., Latif, S., Hamadou, W. S. ., Alreshidi, M. ., Ashraf, S. A. ., Patel, M. ., Humaidi, J. R. ., Anouar, E. H. ., Kadri, A., & Noumi, E. (2024). Green synthesis and characterization of silver nanoparticles from Ducrosia flabellifolia Boiss. aqueous extract: Anti-quorum sensing screening and antimicrobial activities against ESKAPE pathogens: Plant silver NPs and their activities. Cellular and Molecular Biology, 70(2), 88–96. <https://doi.org/10.14715/cmb/2024.70.2.13>.

[55]	Cinteza LO, Scomoroscenco C, Voicu SN, Nistor CL, Nitu SG, Trica B, Jecu ML, Petcu C. Chitosan-stabilized ag nanoparticles with superior biocompatibility and their synergistic antibacterial effect in mixtures with essential oils. Nanomaterials 2018;8. <https://doi.org/10.3390/nano8100826> .

[56]	Keller MR, Dörr T. Bacterial metabolism and susceptibility to cell wall-active antibiotics. Adv Microb Physiol, vol. 83, Academic Press; 2023, p. 181–219. <https://doi.org/10.1016/bs.ampbs.2023.04.002> .

[57]	Fernández-Martínez A., Charlet L. Selenium environmental cycling and bioavailability: a structural chemist point of view. Rev. Environ. Sci. Biotechnol. 2009;8:81–110.

[58]	Zhang S.Y., Zhang J., Wang H.Y., Chen H.Y. Synthesis of selenium nanoparticles in the presence of polysaccharides. Mater. Lett. 2004;58:2590–2594.

[59]	Stepankova H, Michalkova H, Splichal Z, Richtera L, Svec P, Vaculovic T, Pribyl J, Kormunda M, Rex S, Adam V, Heger Z. Unveiling the nanotoxicological aspects of Se nanomaterials differing in size and morphology. Bioact Mater 2023;20:489–500. <https://doi.org/10.1016/j.bioactmat.2022.06.014> .

[60]	Tayel AA, El-Tras WF, Moussa S, El-Baz AF, Mahrous H, Salem MF, Brimer L. Antibacterial action of zinc oxide nanoparticles against foodborne pathogens. J Food Saf 2011;31:211–8. <https://doi.org/10.1111/j.1745-4565.2010.00287.x> .

[61]	Mousavi SM, Hashemi SA, Kalashgrani MY, Omidifar N, Bahrani S, Rao NV, Babapoor A, Gholami A, Chiang WH. Bioactive Graphene Quantum Dots Based Polymer Composite for Biomedical Applications. Polymers (Basel) 2022;14. <https://doi.org/10.3390/polym14030617> .

[62]	Shah M, Fawcett D, Sharma S, Tripathy SK, Poinern GEJ. Green synthesis of metallic nanoparticles via biological entities. Materials 2015;8:7278–308. <https://doi.org/10.3390/ma8115377> .

[63]	Devi L, Kushwaha P, Ansari TM, Kumar A, Rao A. Recent Trends in Biologically Synthesized Metal Nanoparticles and their Biomedical Applications: a Review. Biol Trace Elem Res 2024;202:3383–99. <https://doi.org/10.1007/s12011-023-03920-9> .

[64]	El-Zayat, M.M., Eraqi, M.M., Alrefai, H., El- Khateeb, A.Y., Ibrahim, M.A., Aljohani, H.M. Aljohani, M.M. and Elshaer, M.M., The antimicrobial, antioxidant, and anticancer activity of greenly synthesized selenium and zinc composite nanoparticles using Ephedra aphylla extract. Biomolecules, 11(3), p.470 (2021).

[65]	Gidado MJ, Gunny AAN, Gopinath SCB, Ali A, Wongs-Aree C, Salleh NHM. Challenges of postharvest water loss in fruits: Mechanisms, influencing factors, and effective control strategies – A comprehensive review. J Agric Food Res 2024;17. <https://doi.org/10.1016/j.jafr.2024.101249> .

[66]	Bal E. Postharvest Application of Chitosan and Low Temperature Storage Affect Respiration Rate and Quality of Plum Fruits. <Http://IrJkuatAcKe/Handle/123456789/4380> 2018.

[67]	El-Sayed SM, Nossier MI, Nossier AI. Faba beans with enhanced antioxidant activity ameliorate acetic acid-induced colitis in experimental rats. Food Funct 2022;13:11865–78. <https://doi.org/10.1039/d2fo02782h> .

[68]	Emragi E, Kalita D, Jayanty SS. Effect of edible coating on physical and chemical properties of potato tubers under different storage conditions. LWT 2022;153. <https://doi.org/10.1016/j.lwt.2021.112580> .

[69]	Duan C, Meng X, Meng J, Khan MIH, Dai L, Khan A, An X, Zhang J, Huq T, Ni Y. Chitosan as A Preservative for Fruits and Vegetables: A Review on Chemistry and Antimicrobial Properties. Journal of Bioresources and Bioproducts 2019;4:11–21. <https://doi.org/10.21967/jbb.v4i1.189> .

[70]	Vieira TM, Moldão-Martins M, Alves VD. Composite coatings of chitosan and alginate emulsions with olive oil to enhance postharvest quality and shelf life of fresh figs (*Ficus carica l. cv. ‘pingo de mel’*). Foods 2021;10. <https://doi.org/10.3390/foods10040718> .

[71]	Choudhary P, Jain V. Effect of post-harvest treatments of selenium on physico-chemical quality in guava (*Psidium guajava L.*) fruit. Horticulture International Journal  2018;2. <https://doi.org/10.15406/hij.2018.02.00024> .

[72]	Li H, Huang Z, Addo KA, Yu Y. Evaluation of postharvest quality of plum (Prunus salicina L. cv. ‘French’) treated with layer-by-layer edible coating during storage. Sci Hortic 2022;304. <https://doi.org/10.1016/j.scienta.2022.111310>.

[73]	Massolo JF, Concellón A, Chaves AR, Vicente AR. 1-Methylcyclopropene (1-MCP) delays senescence, maintains quality and reduces browning of non-climacteric eggplant (*Solanum melongena L.*) fruit. Postharvest Biol Technol 2011;59:10–5. <https://doi.org/10.1016/j.postharvbio.2010.08.007> .

[74]	Zhao Y, Zhu X, Hou Y, Pan Y, Shi L, Li X. Effects of harvest maturity stage on postharvest quality of winter jujube (Zizyphus jujuba Mill. cv. Dongzao) fruit during cold storage. Sci Hortic 2021;277. <https://doi.org/10.1016/j.scienta.2020.109778>.

[75]	Díaz-Mula HM, Serrano M, Valero D. Alginate Coatings Preserve Fruit Quality and Bioactive Compounds during Storage of Sweet Cherry Fruit. Food Bioproc Tech 2012;5:2990–7. <https://doi.org/10.1007/s11947-011-0599-2> .

[76]	Tanaka Y, Sasaki N, Ohmiya A. Biosynthesis of plant pigments: Anthocyanins, betalains and carotenoids. Plant Journal 2008;54:733–49. <https://doi.org/10.1111/j.1365-313X.2008.03447.x> .

[77]	Gantait SS, Pal P. Anthocyanin content of spray Chrysanthemum cultivars under polyhouse and open field conditions. vol. 1. 2010.

[78]	Zhu Z, Chen Y, Shi G, Zhang X. Selenium delays tomato fruit ripening by inhibiting ethylene biosynthesis and enhancing the antioxidant defense system. Food Chem 2017;219:179–84. <https://doi.org/10.1016/j.foodchem.2016.09.138> .

**Acknowledgement**

The authors would like to deeply thank Prof. Dr. Mervat Ahmad, Head of Biochemistry Department, Faculty of Agriculture, Ain Shams University for her invaluable support, and the New Programs Administration, for providing all lab. Facilities for microbiological analysis.

**Funding**

Open access funding provided by The Science, Technology & Innovation Funding Authority (STDF) in cooperation with The Egyptian Knowledge Bank (EKB).

**Authors Contribution**

Salwa M. El-Sayed (S.M.E.) conceived the idea, S.M.E. supervised the project, and Mohamed A. Nasser (M.A.N.), Samah H. Abu-Hussien (S.H.A.) co-supervised the project. S.M.E., Hamza T. O. Abdelaziz (H.T.A), Seif Eldin M. Mohamed (S. E.M), Samir K. A. Younis (S. K.Y), Nada Ahmed (N.A.), Mary N. Michaeel (M.N.M.), Mohamed K. Abou El-Nasr (M.K.A.) and M.A.N. conducted the experimental work, while S.M.E., Mahmoud Salah (M.S.) and, M.A.N. analyzed the data. Bahaa Hemdan (B.H.), and, S.H.A. analyzed and interpreted microbiological data, Mahmoud Adel (M.A.), Ashraf Bakry (A.B.), Naglaa M. Ebeed (N.M.E.), S.H.A., B.H., S.M.E., M.S. and M.A.N. wrote and reviewed the manuscript. All authors wrote the draft manuscript and reviewed and edited it. All authors read and approved the manuscript.

**Ethical Approval**

This article does not contain any studies with human participants or animals performed by any of the authors.

**Conflict of Interest**

The authors declare that they have no known competing interests.

**Data availability**

All microorganisms used were obtained from MIRCEN, Cairo, Egypt and were deposited in the following culture collections:

*Staphylococcus aureus* ATCC 29737 was from the ATCC collection <https://www.atcc.org/products/29737>

*Bacillus cereus* ATCC 11778 was from the ATCC collection <https://www.atcc.org/products/11778>

*K. pneumonia* ATCC 700603 was from ATCC collection [*https://www.atcc.org/products/700603*](https://www.atcc.org/products/700603)

*E. coli ATCC 8379 was from ATCC collection* [*https://www.atcc.org/products/8379*](https://www.atcc.org/products/8739)

*Aspergillus niger* ATCC 13496 was from ATCC collection [Aspergillus niger van Tieghem - 13496 | ATCC](https://www.atcc.org/products/13496)

*Candida albicans DSM 1386 was from DSM collection* [*Candida albicans DSM 1386 | Strains | MediaDive*](https://bacmedia.dsmz.de/strains/view/DSM%201386)
